# Physics-Topology-Anchored Learning: A Robust and Lightweight Framework for Time-Series Prediction and Anomaly Detection Under Data Scarcity

**DOI:** 10.3390/s26051721

**Published:** 2026-03-09

**Authors:** Xuanhao Hua, Weiqi Yin, Libin Wang, Meng Ma, Jianfeng Yuan, Jing Zhang

**Affiliations:** 1 School of Future Technology, Xi’an Jiaotong University, Xi’an 710049, China; huaxuanhao@stu.xjtu.edu.cn (X.H.); 3124198004@stu.xjtu.edu.cn (W.Y.); 2 Hangzhou Special Equipment Inspection and Science Research Institute, Hangzhou 310051, China; jiminydz@stu.xjtu.edu.cn (J.Y.); zhujunchaohz@126.com (J.Z.); 3 School of Mechanical Engineering, Xi’an Jiaotong University, Xi’an 710049, China

**Keywords:** fault diagnosis, physics-topology anchored, data scarcity, inductive bias, graph neural networks

## Abstract

Health monitoring of complex systems is critical for ensuring reliability and achieving cost-effective reusability. However, deploying deep learning models in this domain is impeded by two primary constraints: the scarcity of high-quality fault samples and the restricted computational resources available on-board. To address these challenges, this paper proposes a Physics-Topology-Anchored Learning (PTAL) framework. The core innovation lies in the effective integration of physical inductive bias into the model architecture. Specifically, PTAL incorporates a predefined adjacency matrix, derived from the physical mechanism, as a structural prior. This design anchors the neural network to explicit physical causality, effectively constraining the hypothesis space and reducing the model’s dependency on large-scale data. Furthermore, by coupling this physics-informed structure with a lightweight recurrent attention mechanism, the model avoids the high computational overhead typical of generic large-scale networks. Experimental evaluations demonstrate that PTAL achieves a peak diagnostic accuracy of 97.8% and a low standard deviation of 0.1145, significantly outperforming baseline models in data-scarce regimes. The results confirm that the proposed model successfully leverages physical bias to maintain a favorable trade-off between diagnostic performance and computational efficiency, making it highly suitable for the resource-constrained environments of complex systems.

## 1. Introduction

Today, with linking space development to our future lives, reusable liquid rocket engines are the future trend. With considerable efforts being taken worldwide to develop RLREs, it is associated with new challenges. In the field of health-monitoring system (HMS) techniques, it is possible to achieve functional reliability and cost reduction without having to carry out renewed validation tests for each launch [1]. Compared with the conventional expendable type of LREs, the most attractive feature of RLREs is the achievement of drastic price reduction [2]. Thus, the increase in reliability and safety of RLREs is needed to avoid catastrophic accidents. In addition to improving reliability at design and manufacturing stages, it is needed to develop a fault-tolerance system, which means an engine is capable of detecting its fault intelligently and being stopped immediately and safely when a fault occurs during flight [3]. This procedure is usually achieved through developing advanced HMS techniques. As launch vehicle reusability becomes commercial, HMS is becoming essential and develops as systems age [4].

During the mission assurance process, collected telemetry from the whole vehicle system is continuously monitored in the pre-launch, launch and return stages, which is achieved through several thousand sensors [5]. To detect the imminent or potential failure in a mission process in real time, HMS techniques traditionally relied on a redline cutoff system, which is calculated by comparing redline limits and historical data [6]. However, this method relies on the historical data and ignores the difference between different engines. Thus, there is a need to develop advanced algorithms for anomaly detection and fault isolation.

The methods of fault detection and isolation for engines are generalized into three categories: rule-based methods, model-based methods and data-driven methods [7]. Rule-based methods carried out fault diagnosis according to rules collected from the knowledge of not only experienced engineers and large historic hot-firing testing data but also system fault simulation results [8]. The rule-based methods require significant experience with known system failure modes, which have difficulty handling novel behavior, and become overly cumbersome to manage with an increase in system complexity [9]. Model-based methods take advantage of physical laws within complex systems to build the relationship between input parameters and output performances [10]. The advantages lie in its interpretability through known scientific principles. By providing a baseline model for underlying physical relations, the parameters could be determined through filtering algorithms with historical data. For example, multi-physics system-level modeling and simulation for a target system were conducted in [11], which adopted fault diagnostics in several different physical domains and components. However, it is challenging to build accurate models due to the inherent complexity of the physical processes [12]. While rule-based and model-based methods offer high interpretability, their reliance on exhaustive expert knowledge or perfectly accurate physical equations makes them brittle when facing the high-frequency dynamics and nonlinear coupling characteristic of RLRE startup transients. Data-driven methods utilize monitoring data to detect and isolate potential faults with machine learning algorithms or statistical models, which could remedy the challenges of model-based methods, which rely on prior knowledge about the system [13]. More attention is paid to data-driven methods since they only depend on monitoring data without understanding the physical model of the system.

Machine learning methods have been widely investigated in fault diagnosis due to the recent breakthroughs of deep learning algorithms [14]. For instance, Park et al. [15] proposed a fault diagnosis method based on CNNLSTM. A numerical simulation case study was carried out to demonstrate its performance. The results showed it can improve the classification accuracy compared to conventional red-line cutoff systems. Li et al. [16] combined soft target difference scaling and metric learning to calculate the engine state discrimination threshold. The measured data collected from liquid rocket engines were used to verify its performance. Zhu et al. [17] presented a fault detection method based on a convolutional auto-encoder and a one-class support vector machine for the steady-state process of LREs. The experimental results demonstrate its advantages compared with the traditional redline system, adaptive threshold algorithm, and back-propagation neural network. However, these data-driven approaches have notable drawbacks: the CNN-LSTM model lacks physical grounding and risks overfitting under data scarcity; the metric learning method ignores the structural connectivity of engine components; and the auto-encoder approach is primarily optimized for steady-state processes rather than the complex causality of startup transients.

Generally speaking, data-driven and model-based methods both have their own characteristics and could suffer from certain deficiencies when applied to complex engineering problems in fault diagnosis [18]. However, in the specific domain of RLREs, the challenges are even more pronounced. While state-of-the-art deep learning models (e.g., Transformers) have achieved remarkable success in general fields, their deployment in RLRE health monitoring is severely impeded by two primary constraints: the inherent scarcity of high-quality fault samples and the restricted computational resources available on-board. Generic large-scale models often suffer from overfitting when training data is limited and impose computational burdens that exceed the capacity of edge devices.

To address these challenges, there is a growing consensus that solutions require novel paradigms that effectively integrate physical inductive bias with lightweight deep learning architectures [19]. In this study, a Physics-Topology-Anchored Learning (PTAL) framework is proposed. Unlike purely data-driven approaches that attempt to learn latent relationships from scratch, PTAL utilizes the engine’s numerical mechanism to construct a predefined physical adjacency matrix as a structural prior. This design anchors the learning process to explicit physical causality. Furthermore, instead of computationally intensive self-attention layers, we employ lightweight recurrent layers to model the temporal inertia of fluid dynamics, combined with a focused attention mechanism for feature weighting. By this means, the model extracts spatiotemporal features efficiently, maintaining a balance between diagnostic performance and model complexity suited for RLREs.

The main contributions of this paper are as follows:

i. We propose a novel framework specifically designed for the robust fault diagnosis of RLREs under data scarcity. By encoding the intrinsic component connectivity and fluid paths into the model as a “hard” structural constraint (i.e., physical inductive bias), PTAL reduces the hypothesis space. This “anchoring” effect significantly enhances the model’s robustness and generalization ability compared to purely data-driven “black-box” methods.
ii. Aiming to overcome the computational bottlenecks of deploying deep learning on aerospace edge devices, we design a lightweight recurrent graph architecture. Instead of learning a dense adjacency matrix from data or using heavy Transformer blocks, the relationship graph is deterministically determined by the system’s physical mechanism, and sequential features are captured by efficient recurrent units. This design circumvents the high parameter redundancy of large-scale models, ensuring operational efficiency.
iii. The performance of the proposed PTAL method is verified through extensive simulation and real-world experiments. The results demonstrate that the model achieves competitive diagnostic accuracy even in few-shot scenarios and maintains a favorable trade-off between accuracy and computational cost, confirming its potential for practical, resource-constrained RLRE-health-monitoring tasks.

The rest of this paper is organized as follows. Section 2 introduces the proposed framework for fault diagnosis. In Section 3, we illustrate the performance of the proposed method utilizing simulation and practical monitoring data. In Section 4, we compare our approach against other methods. Finally, some remarkable conclusions are drawn in Section 5.

## 2. Methodology

This study aims to achieve two primary objectives tailored for the health monitoring of complex engineering systems such as RLREs. The first objective is to effectively mitigate the challenge of data scarcity by embedding physical inductive bias directly into the learning process. Specifically, we seek to replace data-hungry correlation learning with a deterministic physics-topology-anchored approach. Simultaneously, we aim to establish a lightweight and interpretable diagnostic module that comprehensively captures both the temporal dynamics of the system and the spatial causality among components. This dual focus ensures robust performance and operational efficiency, even when deployed on resource-constrained platforms.

### 2.1. Architecture

**Figure 1:**
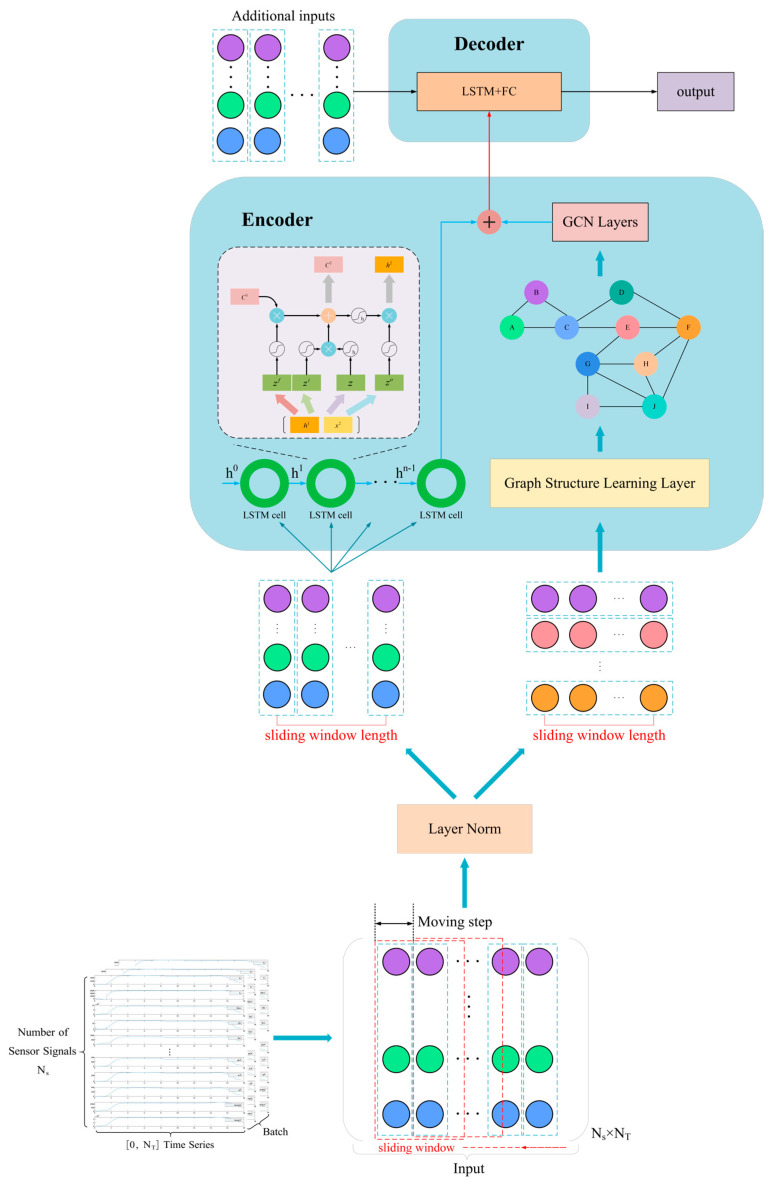
The structure of the proposed model.

The overall architecture of the proposed Physics-Topology-Anchored Learning (PTAL) framework, as illustrated in Figure 1, employs a specialized physics-informed encoder–decoder structure designed to map multi-dimensional sensor data into a physically meaningful latent space. The physics-anchored encoder serves as the core component for extracting spatiotemporal features by utilizing a hybrid architecture that integrates graph convolutional networks (GCNs) and long short-term memory (LSTM) units. In terms of spatial modeling (topological anchoring), the GCN layers are constrained by a predefined physical adjacency matrix rather than learning a fully connected graph from scratch. This matrix encodes the intrinsic inter-component connectivity, effectively forcing the model to aggregate information solely along physically causal pathways. In parallel, the LSTM units capture the dynamic evolution and temporal dependencies of the monitoring parameters. Subsequently, the outputs from the physics-constrained GCN and the temporal LSTM are fused to form a compact latent state, which encapsulates the system’s health status while filtering out irrelevant noise. Following this, the Diagnostic Decoder interprets the latent features for the final diagnostic task. Comprising corresponding LSTM layers followed by fully connected (FC) layers, the decoder maps the high-level representation—enriched with physical topology information—to the target output space to generate the final fault classification result.

### 2.2. About Dataset and Prior Knowledge

The experimental data is derived from a high-fidelity physical simulation of a liquid rocket engine, capturing the startup transient phase under both normal ignition and three distinct fault modes. From the original 28 monitoring variables (excluding timestamps), a subset of 10 critical features was selected for model training based on their physical significance. These features include main combustion chamber pressure (Pc), main combustion chamber temperature (Tc), fuel preburner chamber pressure (fpbPc), low-pressure fuel turbopump outlet pressure (lpftpPo), low-pressure fuel turbopump speed (lpftpN), low-pressure fuel turbopump flow rate (lpftpQ), high-pressure oxidizer turbopump outlet pressure (hpftPo), high-pressure oxidizer turbopump speed (hpftpN), high-pressure oxidizer turbopump flow rate (hpftpQ), and nozzle throat cooling liquid pressure (ncP) for model learning. Furthermore, the causal relationships among these parameters were established through physical mechanism analysis and incorporated as structural prior knowledge. The resulting physical adjacency matrix is presented in Table 1.

### 2.3. Long Short-Term Memory for Time Dependencies

RNNs are extensively employed in domains such as natural language processing [20,21] and time-series prediction [22,23], owing to their distinctive data propagation structure and exceptional capability in handling temporal dependencies within sequential data. Long short-term memory (LSTM), a specialized variant of recurrent neural networks, excels at capturing and encoding long-range dependencies in time-series data [24]. Consequently, in this study, we utilized LSTM to model and capture the temporal dependencies within the data.

Considering the characteristics of the dataset used, it consists of time-series data where each sample point is composed of different sensor variables at the same time point. The entire network propagates data along the time axis. Therefore, we use LSTM to handle temporal dependencies, as all data points are distributed along the time axis. Each data point represents the values of all features considered at the same time step. The general computation flow for the LSTM time step t is as follows.

**Figure 2:**
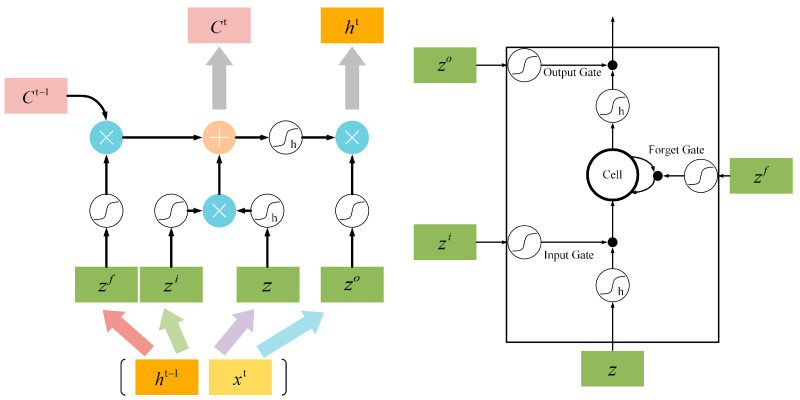
A block inside an LSTM network.

As shown in Figure 2, the temporal features of the engine startup transients are captured via an LSTM layer.

After calculation of Equations (1)–(3), the LSTM unit under the current time step obtains two output vectors $C^{t}$ and $h^{t}$, and passes them into the LSTM unit of the next time step.

(1) $$\left[\begin{array}{c} \begin{array}{c} z^{f}\\ z^{i} \end{array}\\ \begin{array}{c} z^{o}\\ z \end{array} \end{array}\right]=\left[\begin{array}{c} \begin{array}{c} W_{f}\\ W_{i} \end{array}\\ \begin{array}{c} W_{o}\\ W \end{array} \end{array}\right]\cdot \left[\begin{array}{c} h^{t-1}\\ X^{t} \end{array}\right]+\left[\begin{array}{c} \begin{array}{c} b_{f}\\ b_{i} \end{array}\\ \begin{array}{c} b_{o}\\ b \end{array} \end{array}\right]$$

(2) $$C^{t}=sigmoid\left(z^{f}\right)\;⨀\;c^{t-1}+sigmoid\left(z^{i}\right)\;⨀\;sigmoid\left(z\right)$$

(3) $$h^{t}=sigmoid\left(z^{o}\right)\;⨀\;\mathrm{tan h}\left(C^{t}\right)$$

LSTM performs internal calculations and data propagation through input gates, forget gates, and output gates. The sigmoid function allows LSTM units to selectively remember and forget intermediate data. In summary, LSTM units encode multi-dimensional time-series data, extracting temporal dependencies, and find wide applications in multi-sensor fault detection problems.

### 2.4. Graph Neural Network

#### 2.4.1. Physics-Embedded Graph

**Figure 3:**
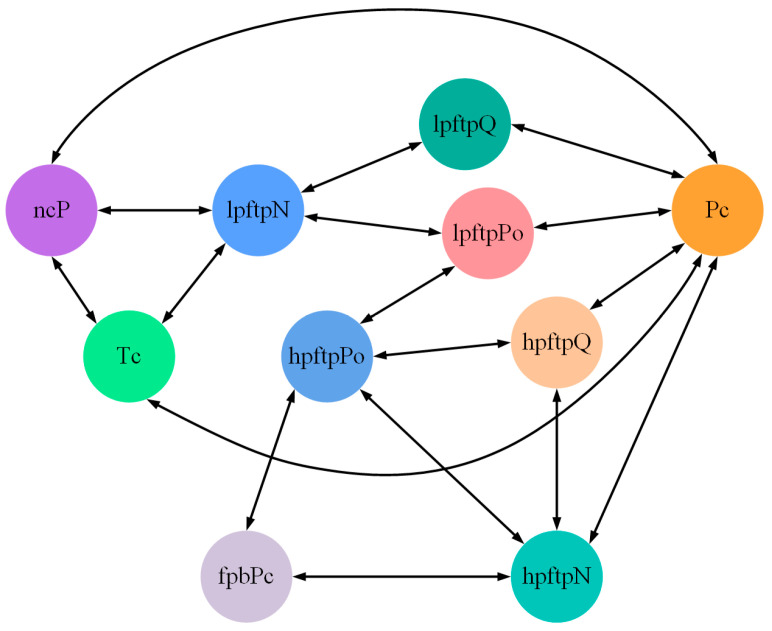
Physics-embedded sensor graph of the RLRE fuel system.

A graph consists of a finite non-empty set of vertices and a set of edges between the vertices [25]. GNN’s typical input comprises two main components: the graph structure and node labels. Based on the context above, the structure of the graph will be input to the model in the form of an adjacency matrix for learning or iteration. Since graph networks excel at capturing spatial correlations in data, we consider each sensor channel as a graph node, and at each time step, each sensor can represent a node in the graph network. Given that the experimental data in this study consists of transient data from liquid rocket engine startups, there are clear graph relationships between sensor sequences. Therefore, in this research, the graph structure depicted in Figure 3 is qualitatively defined based on the data presented in Table 1. Specifically, the graph structure represents the actual physical model [26], namely a physics-embedded graph.

#### 2.4.2. Graph Convolutional Network (GCN)

The concept of GCN was first introduced by Thomas Kipf et al. in [27]. Graph data is widely prevalent across various domains such as social networks, recommendation systems, bioinformatics, knowledge graphs, and more. These datasets typically exhibit complex topological structures and relationships between nodes, making traditional machine learning methods often ineffective for handling them. Hence, there is a demand for the development of new deep learning models to tackle this unstructured graph data. Simultaneously, CNNs have achieved tremendous success in computer vision by effectively capturing local features and spatial relationships in images. Given that graphs also possess locality and spatial structure (in the form of relationships between nodes), researchers began exploring the application of CNN principles to graph data to enhance their modeling capabilities [28].

The introduction of GCN has filled the void in graph data modeling and provided a powerful deep learning framework for handling graph data [29]. It achieves this by applying convolutional operations to graph data, allowing the model to facilitate information propagation between nodes, thereby enabling the learning of node representations and relationships among them. In contrast to the previous section, we will no longer impose a predefined graph structure, meaning that we will not provide any adjacency matrix or internal relationships. Instead, in this study, these relationships will be learned by GCN itself, aiming to uncover potential, unnoticed connections among various sensor channels. The formula for the graph learning layer is as follows:

(4) $$L_{1}=\mathrm{tan h}\left({Embedding}_{1}\left(idx\right)\Theta_{1}\right)$$

(5) $$L_{2}=\mathrm{tan h}\left({Embedding}_{1}\left(idx\right)\Theta_{2}\right)$$

(6) $$A=\mathrm{ReLU}\left(\mathrm{tan h}\left(L_{1}{L_{2}}^{T}\right)\right)$$

where *embeddings* represent two distinct randomly initialized layers that transform scalar index vectors into embedding matrices. In this context, the variable *idx* in the equation denotes node indices, which are positive integers representing node identifiers. Since the embedding layers are trainable, the adjacency matrix will also change as the model iterates, indicating that this model can be used for learning graph structures. $\Theta_{1}$ and $\Theta_{2}$ are the model parameters of two simple feedforward neural networks.

Essentially, the ultimate learning objective and output form are the hidden vector representations of each node in the graph. The graph convolution module aims to combine a node’s information with that of its neighbors to handle spatial dependencies within the graph. In other words, node representations are created by transforming representations of neighboring nodes. The current features of different nodes are reflected in the hidden layer H, with specific dimensions determined by the network’s construction. The next-step feature vector of the target node is calculated based on its current features and the features of its neighboring nodes. In general, a graph convolutional network computes features for all nodes simultaneously. However, before entering the first graph convolutional layer, it is typically necessary to have initial features for each node.

In this example, the initial features are derived from time-series data of different variables. The specific computation method is as follows:

(7) $$H^{\left(0\right)}=X$$

(8) $$\tilde{A}=A+I$$

(9) $$H^{\left(l+1\right)}=f\left(H^{\left(l\right)},A\right)=\sigma \left({\tilde{D}}^{-\frac{1}{2}}\tilde{A}{\tilde{D}}^{-\frac{1}{2}}H^{\left(l\right)}W^{\left(l\right)}\right)$$

Equation (7) represents that the initial features of each node are derived from the processed input data. Equation (8) signifies that the computation of node features also takes into account the node’s own information, effectively adding a self-loop to each node. Equation (9) represents the network’s propagation function, where the matrix D refers to the diagonal matrix, with values on the diagonal indicating the number of direct neighbors for each node. It normalizes the matrix A to ensure the stability of model training.

This propagation scheme can be understood as a stepwise refinement of a simple neighbor aggregation operation. Starting from the raw adjacency matrix A, a self-loop is first added ($\tilde{A}=A+I$) to retain the node’s own features. To prevent nodes with high degrees from dominating the aggregation, symmetric normalization is then applied: $\hat{A}={\tilde{D}}^{\left(-1/2\right)}\tilde{A}{\tilde{D}}^{\left(-1/2\right)}$, where $\tilde{D}$ is the degree matrix of $\tilde{A}$. The final operation $\hat{A}H^{\left(l\right)}W^{\left(l\right)}$ computes a degree-normalized weighted average of each node’s features and those of its physically coupled neighbors. This ensures numerical stability and scale invariance, which is especially important when stacking multiple GCN layers.

It is important to note that, unlike the graph learning layer described in Equations (7)–(9) (which is employed only for the data-driven baseline in Section 3.3), the PTAL framework does not learn the adjacency matrix. Instead, we adopt the fixed physical adjacency matrix defined in Section 2.2 and apply the standard GCN propagation as above. This fixed topology acts as a hard structural inductive bias, constraining information flow to physically causal paths and reducing overfitting under data scarcity.

**Figure 4:**
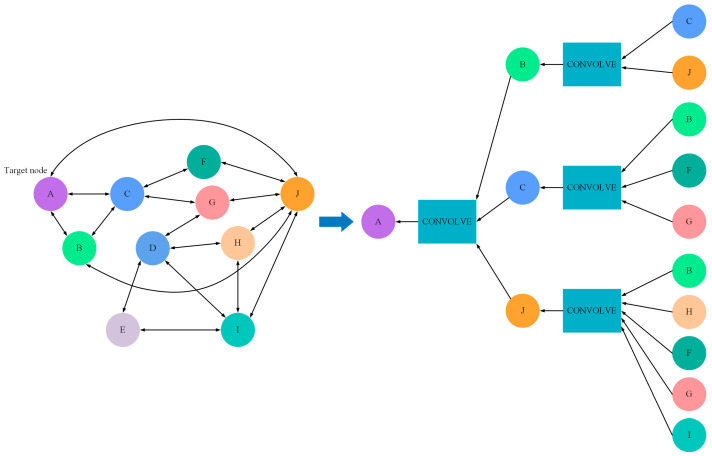
Example of stacking methods for GCNs.

In Figure 4, the left section illustrates the graph architecture, while the right section represents an example of two layers of GCN information flow. In this scenario, the information aggregated for the target node A is much more extensive compared to what a single-layer graph convolutional network would aggregate. However, too many convolutional layers can cause the computation to increase exponentially, implying that it is generally advisable to use only two to three graph convolutional layers [30].

### 2.5. The Proposed PTAL-Attention Architecture

Attention models have seen widespread adoption in various domains of deep learning, appearing prominently in tasks spanning image processing, speech recognition, and various types of tasks in natural language processing. In [31], Mnih et al. proposed the use of the attention mechanism in conjunction with RNN models for image classification, yielding impressive performance results, which ignited the popularity of attention mechanisms. The attention mechanism has been widely employed in various deep learning tasks based on RNN models [32].

**Figure 5:**
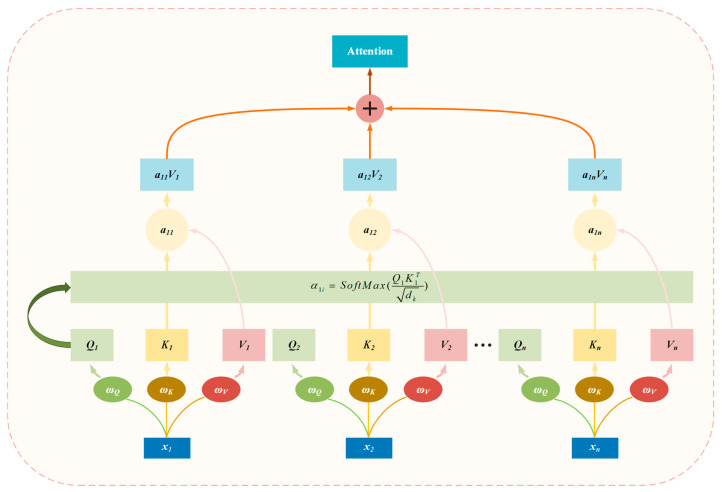
Attention concept used in LSTM and GNNs.

The attention mechanism posits that different features in a network have varying degrees of importance, with later layers focusing more on crucial information while suppressing less important details [33]. The fundamental components of attention include three parts: query, key, and value. Typically, once these three components are defined, the computation 97 of attention becomes straightforward. The concept of attention is illustrated in Figure 5. Generally, in each attention computation, there is a single query and multiple pairs of keys and values. In Equation (10), the query first obtains the current key’s score by inputting it together with the scoring function and then transforms the scores into probability weights using the SoftMax function. There are various options for scoring functions in attention mechanisms, including addition [34], dot product [35], scaled dot product [36], and more. Here, we use self-attention as an example [37].

The final attention value is determined by the weighted sum of the original values and the scoring function, as shown in Equations (11) and (12).

(10) $$\alpha_{1i}=softmax\left(\frac{Q_{1}K_{i}^{T}}{\sqrt{d_{k}}}\right)$$

(11) $$Attention\left(Q_{1},K,V\right)=\sum_{i=1}^{d_{k}}\alpha_{1i}V_{i}$$

(12) $$Attention\left(Q,K,V\right)=softmax\left(\frac{QK^{T}}{\sqrt{d_{k}}}\right)V$$

Let Q be of dimensions M×d, where M is the length of the source time-series data; key (K) has dimensions N×d, where N is the length of the target time-series data, and d represents the vector dimensionality. It is evident that vectors Q and K are of equal length because they operate in the same vector space and require similarity comparisons, i.e., dQ=dK=d. However, V has dimensions N×dv, and its vector length can differ from Q and K. One can consider the key-value pairs of K and V to represent the same data in different state spaces. Consequently, the final attention values form an M×dv matrix. After applying Softmax weighting, weight coefficients are obtained, and based on these coefficients, a weighted summation is performed across the dv dimensions, resulting in M attention values, each with a dimensionality of dv. It is worth noting that a scaling factor is introduced in the formula primarily for normalization purposes. This helps decouple the steepness of the Softmax distribution from d, ensuring gradient stability during training.

Therefore, by employing the aforementioned approach, as long as the elements of the attention mechanism are defined within LSTM and GNN, the attention mechanism can be easily applied to both of these networks.

**Figure 6:**
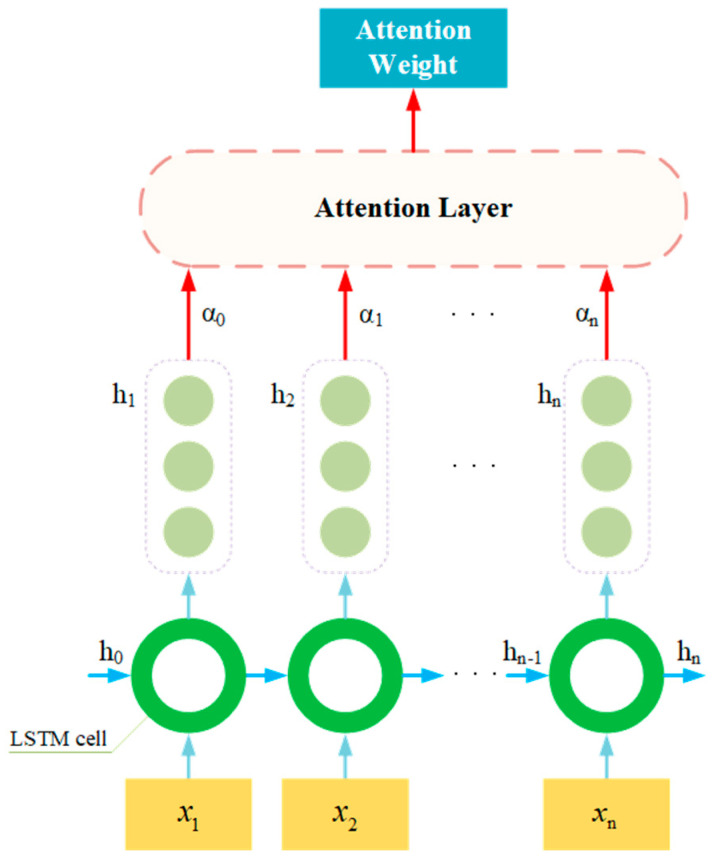
Application of attention in LSTM.

While LSTM performs better than regular recurrent neural networks in addressing the vanishing gradient problem and capturing long-term dependencies in data, information loss during the encoding process still poses a significant challenge, primarily due to the presence of forget gates. In Figure 6, the introduction of attention mechanisms allows the model to automatically focus on critical parts of the time-series while ignoring less important aspects. This enhances the model’s attentional focus, enabling it to better capture meaningful information for the task. Additionally, it helps us understand the basis for the model’s decisions.

Specifically, the interaction between the recurrent temporal modeling and the attention mechanism is designed as a jointly conditioned process. Rather than operating independently, the attention weights are computed based on the sequence of hidden states $H=\{h_{1},h_{2},\ldots ,h_{n}\}$ generated by the LSTM units. This ensures that the importance of each feature is evaluated within the evolving temporal context of the system. During the training phase, the entire architecture is optimized end-to-end via a unified loss function, allowing the gradients to flow back through both the attention layers and the LSTM gates simultaneously. This synergistic optimization enables the model to effectively learn temporal dependencies while prioritizing critical fault signatures.

**Figure 7:**
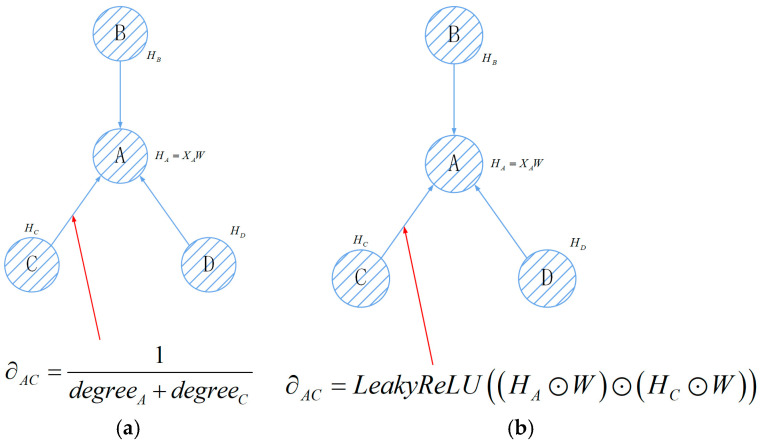
The difference in attention in graph neural networks: (**a**) graph neural network score; (**b**) graph neural network with attention.

When graph recurrent neural networks are combined with attention, the model can iteratively learn to update the edge weight information within the given graph structure, quantitatively determining the degree of correlation between different sensor channels. This provides a more accurate approach to problem modeling. Figure 7 illustrates the before and after of incorporating the attention mechanism into graph neural networks.

### 2.6. Computational Complexity Analysis

This subsection analyzes the computational efficiency of PTAL from both the spatial (graph operations) and temporal (sequence modeling) perspectives. Table 2 summarizes the computational complexity of the key components, providing a quantitative comparison between PTAL and other mainstream architectures.

Spatial complexity (graph operations). In standard GCNs or learned-graph models (e.g., LSTM-GAT), learning a dense adjacency structure typically introduces $O(N^{2})$ parameters and message-passing operations, where N denotes the number of sensors/nodes. PTAL instead adopts a fixed physical adjacency matrix derived from the system topology. As a result, spatial aggregation is performed only along physical connections, leading to O(E) complexity, where E is the number of physical edges. Since RLRE-monitoring systems are usually sparse ($E<<N^{2}$), this setting substantially reduces the spatial computational burden.

Temporal complexity (sequence modeling). Transformer-style self-attention is commonly associated with $O(L^{2})$ complexity with respect to the sliding window length L, which can become expensive for long windows. PTAL uses a recurrent temporal backbone with a focused attention-based weighting mechanism, whose per-window computation scales approximately as O(L). This design keeps temporal modeling cost linear in the window length while retaining the ability to emphasize informative features.

## 3. Experiments

### 3.1. Data Processing

**Figure 8:**
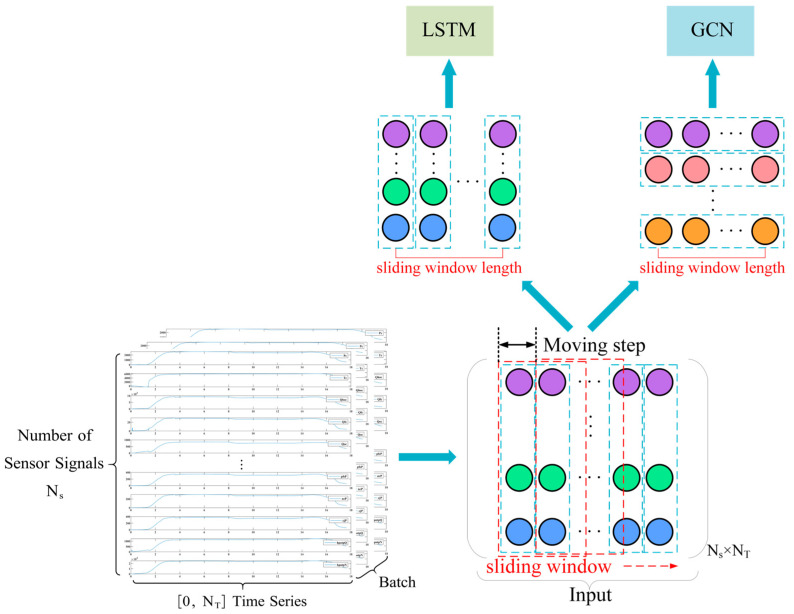
Data processing.

As shown in Figure 8, to align the raw sensor signals with the input requirements of the PTAL framework, we performed rigorous data processing across both temporal and spatial dimensions.

#### 3.1.1. Temporal Segmentation (Sliding Window)

As LSTM requires processing data along the time dimension, we adopt a sliding window mechanism [38,39] to partition the data, as shown by the red dashed boxes in Figure 8. Here, the moving step represents the distance the window moves at each step, depicted by the black dashed line in the figure.

This approach allows us to obtain multiple sets of LSTM sample data with dimensions [time steps, number of sensors]. By adjusting the size of the moving step, we can control the total number of samples obtained. Finally, all processed time step data are adjusted to a three-dimensional tensor of size [batch, time step, feature] and input into the LSTM network.

#### 3.1.2. Spatial Feature Alignment

For the spatial component, the graph topology is explicitly defined by the physical adjacency matrix described in Section 2.3. To ensure spatiotemporal consistency, the definition of node features must strictly align with the temporal segments. We utilize the same data segments obtained from the sliding window to construct the input for the graph neural network (GNN). Specifically, at any given time step within the window, the vector of sensor values serves as the attribute set for the corresponding nodes in the graph. This unified sampling strategy ensures that both the temporal (LSTM) and spatial (GNN) modules process synchronized information, effectively balancing the feature extraction focus between system dynamics and topological dependencies.

### 3.2. Experimental Settings

#### 3.2.1. Dataset Setting and Evaluation Criteria

The dataset in this study is segmented into time periods. There are approximately 400 samples in total, each having dimensions (29, 20,000). The dataset is split into a 70% training set and a 30% validation set.

To evaluate predictive performance, we use accuracy as the primary metric and additionally report macroaveraged precision (Macro-P), recall (Macro-R), and F1-score (Macro-F1) to better reflect false alarms and missed detections in the multi-class diagnosis setting. All results are obtained from 10 independent runs with different random seeds and reported as mean ± standard deviation. For transformer-family baselines, we follow a unified and fair protocol: all models use the same input window length and the same train/validation/test split and are trained with the same optimizer settings and early stopping strategy unless otherwise specified. To substantiate the lightweight claim, we further report the number of trainable parameters (Params) and floating-point operations per input window (FLOPs/window) and summarize the performance–complexity trade-off in Section 4.4.

#### 3.2.2. Hyperparameters

During the training process, we use the Adam optimizer [40] to adjust the learning rate and smooth the gradient update direction, and the loss function employed is the CrossEntropy loss function from PyTorch 2.4 for gradient descent. The initial learning rate is set to 0.001, and the weight decay is 0.0001. To reduce computational time for data processing [41], the sliding window size was fixed to match the duration of a single fault cycle. This structural design ensures that the model focuses on capturing the complete evolution of a fault event within a single processing window.

### 3.3. Baseline Models and Comparative Strategy

**Figure 9:**
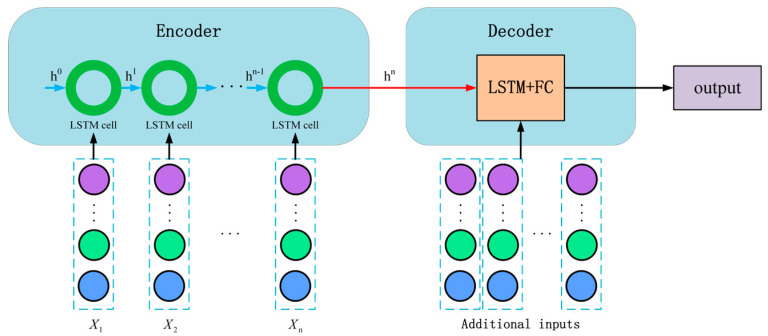
The structure of the LSTM-LSTM&FC model.

To rigorously quantify the contribution of each component in the proposed PTAL framework—namely, the temporal attention, spatial topology, and physical anchoring—we conducted a comparative study against three established encoder–decoder architectures [42]. The selection rationale and structural characteristics of these baselines are detailed below:

- LSTM-LSTM&FC model: As the foundational baseline, this model adopts a classic sequence-to-sequence structure, as illustrated in Figure 9. It comprises a single LSTM layer for encoding temporal features and a corresponding LSTM layer followed by two fully connected (FC) layers for decoding.

This model serves as the “Temporal-Only” control group. Since it treats multi-sensor data as a flat vector and lacks both attention mechanisms and graph topology, it provides a lower-bound performance reference. Comparing PTAL against this baseline highlights the net performance gain achieved by introducing spatiotemporal modeling capabilities.

**Figure 10:**
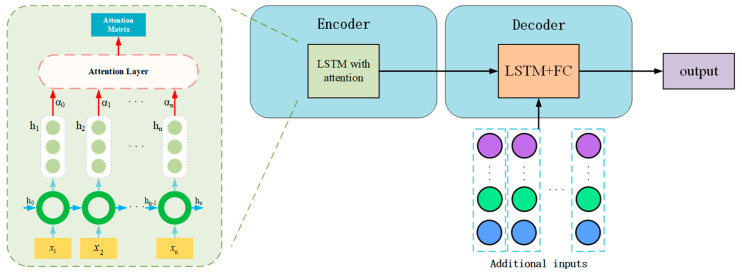
The structure of LSTM with the attention–LSTM&FC model.

**Figure 11:**
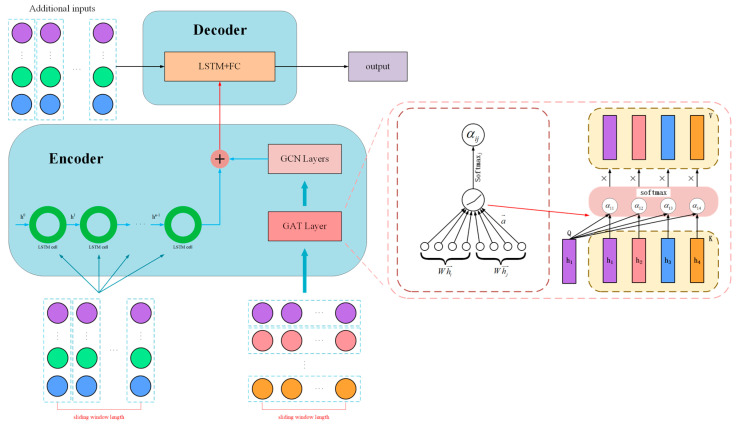
Replace the graph structure learning layer with the GAT layer.

- LSTM with the attention–LSTM&FC model: To assess the impact of temporal focus, this baseline augments the standard LSTM-ED with a temporal attention mechanism, as depicted in Figure 10. This mechanism assigns dynamic weights to different time steps in the input sequence.
- This model is selected to verify the necessity of temporal interpretability. By analyzing the attention weights, we assess whether the model can correctly prioritize time steps containing early fault signatures while disregarding irrelevant noise, thereby validating the efficiency of the attention module in reducing computational redundancy.
- LSTM&GAT-LSTM&FC model: To incorporate spatial dependencies without physical constraints, this model integrates a Graph Attention Network (GAT) layer into the encoder, replacing the physics-anchored graph layer of our proposed method (see Figure 11). Unlike PTAL, which uses a fixed physical matrix, this model attempts to learn the graph structure and edge weights purely from the training data.
- This comparison is critical for validating the superiority of physical inductive bias. By contrasting PTAL with this fully data-driven GAT, we aim to demonstrate that under data-scarce conditions, a predefined physics-topology anchor is significantly more robust and accurate than a graph structure learned from scratch, which is prone to overfitting.

## 4. Results and Discussion

### 4.1. Verification of Physical Consistency via Temporal Attention

In safety-critical aerospace applications, a “black-box” model that achieves high accuracy is insufficient; it must also demonstrate that its decision-making logic aligns with the physical mechanism. To verify this physical consistency, we analyzed the temporal attention weights learned by the PTAL framework. This analysis aims to confirm that the model’s high diagnostic accuracy stems from capturing valid fault signatures rather than overfitting to transient startup noise.

**Figure 12:**
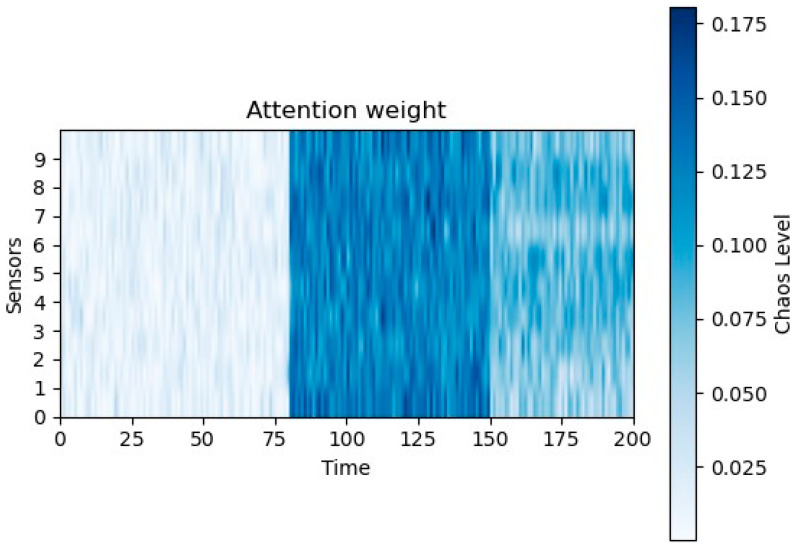
Attention weight.

Figure 12 illustrates the distribution of these attention weights across the time axis within a single fault cycle. To ensure statistical significance, the weights were averaged over multiple test samples and smoothed over 1000-step intervals.

As illustrated in the figure, the attention weights in the initial phase (0~8 s) remain at a near-zero level, maintaining a baseline of approximately 0.025. Physically, this period corresponds to the propellant filling and ignition transient, a phase characterized by high-frequency hydraulic impact and combustion instabilities. It is important to note that the model was not explicitly programmed to ignore this data segment. Instead, through the physics-anchored learning process, the network autonomously determined that the feature gradients in this high-entropy interval do not effectively contribute to differentiating fault modes. This phenomenon demonstrates the model’s intrinsic robustness against transient interference, ensuring that diagnostic decisions are not corrupted by the randomness inherent in the engine startup process.

Subsequently, a distinct transition occurs after t=8 s, where the attention weights exhibit a sharp upward trend, peaking at over 0.150 (a 6-fold increase) during the 15~20 s interval. This transition precisely aligns with the thermodynamic stabilization point of the RLRE and the ground-truth fault injection timestamp. The correspondence between the weight distribution and the physical state indicates that PTAL has successfully learned to identify the fault manifestation boundary—the critical moment when the cumulative deviation of sensor parameters statistically separates from the normal baseline. Consequently, the temporal attention distribution serves as a validity check, confirming that the model exhibits a dynamic alignment with the engine’s physical operation timeline. By focusing computational resources only when the signal-to-noise ratio (SNR) of the fault signature becomes significant, the proposed framework validates its reliability for practical on-board deployment.

### 4.2. Overall Diagnosis Result

The statistical results presented in Table 3 indicate that incorporating spatial dependency modeling improves multi-class diagnosis compared with temporal-only baselines, as graph-based methods (LSTM-GAT and PTAL) consistently outperform LSTM-based encoders without explicit topology. Among all compared approaches, the Transformer-family baselines achieve strong performance, and FEDFormer attains the highest accuracy. Notably, PTAL achieves competitive performance while exhibiting the lowest standard deviation across 10 runs, suggesting improved robustness under data-scarce training. Combined with the efficiency analysis in Section 4.4, these results support that physics-topology anchoring enables a favorable performance–complexity trade-off without relying on heavy architectures.

**Figure 13:**
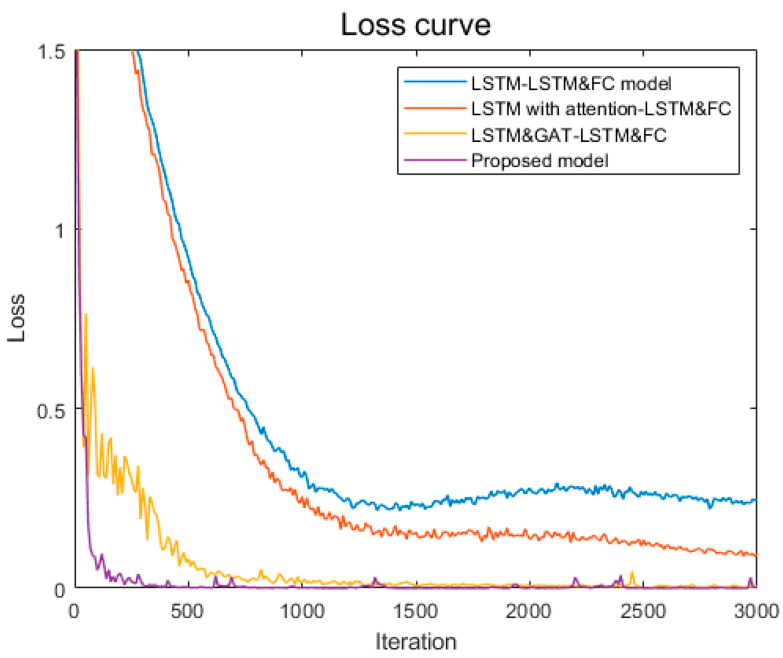
Loss curve.

To verify the statistical significance of the results, we conducted two-sample t-tests comparing PTAL with each baseline model. As shown in Table 3, the *p*-values for all comparisons are less than 0.001, indicating that the performance differences are statistically meaningful. This demonstrates that the integration of physics-topology anchoring provides consistent and robust diagnostic improvements. The loss curve is shown in Figure 13.

### 4.3. Spatial Interpretable Results

This section analyzes the spatial dependencies captured by the models to verify whether the learned correlations align with the physical mechanisms of the RLRE.

#### 4.3.1. Results of the LSTM&GAT-LSTM&FC Model

The LSTM-GAT baseline attempts to autonomously learn the graph structure from the data without physical constraints. Table 4 presents the edge weights learned by this model. The results indicate that specific node pairs, such as B–C, E–D, and E–I, exhibit strong correlations, which aligns with basic domain knowledge. However, a critical observation is that the model assigns non-zero weights between almost all sensor nodes, resulting in a dense and entangled graph structure. While this confirms that effective fault diagnosis requires analyzing the holistic interrelationships between sensor channels, it also reveals a significant limitation: without physical constraints, data-driven models struggle to distinguish between true causal coupling and spurious statistical correlations, leading to a “noisy” interpretation of the system state.

#### 4.3.2. Results of Our Proposed Model

**Figure 14:**
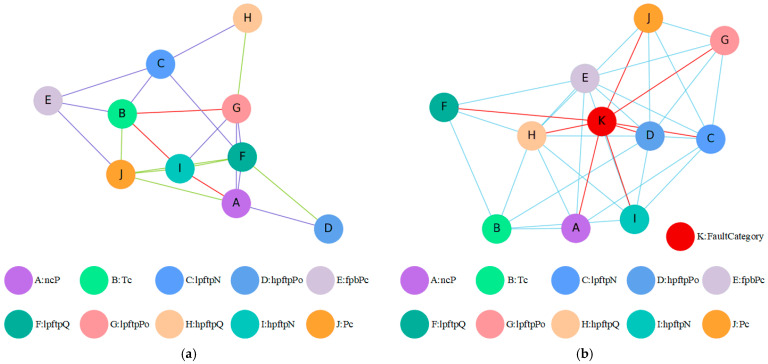
The graph architecture learned by the model we proposed: (**a**) The graph architecture learned by our model_1. (**b**) The graph architecture learned by our model_2.

In contrast to the baseline, the PTAL framework introduces spatial dependencies by applying a Graph Attention Network (GAT) over a physics-anchored topology. Instead of learning the existence of edges from scratch, PTAL learns the attention coefficients for physically valid connections via gradient descent. This process quantifies the strength of influence between coupled components under specific operating conditions, effectively representing the neural network’s understanding of the spatial relationships within the given batch. To facilitate visualization and reveal dominant coupling paths, we pruned edges with attention weights close to zero. The resulting weighted graph structure is depicted in Figure 14a. Compared to the static physical schematic shown in Figure 3, the graph learned by PTAL highlights the dynamic intensity of spatial correlations, revealing latent dependencies that drive fault evolution. Furthermore, to explicitly trace the sensitivity of different sensors to specific failure modes, we constructed an augmented visualization graph, as shown in Figure 14b. In this representation, fault types are introduced as auxiliary nodes. To aid in identifying the root cause, the connections between sensor channels and fault nodes are highlighted in red. These high-weight paths indicate the sensor variables that most significantly contribute to the identification of a specific fault, thereby providing a transparent explanation for the diagnostic decision-making process of the PTAL framework.

### 4.4. Efficiency and Performance–Complexity Trade-Off

**Figure 15:**
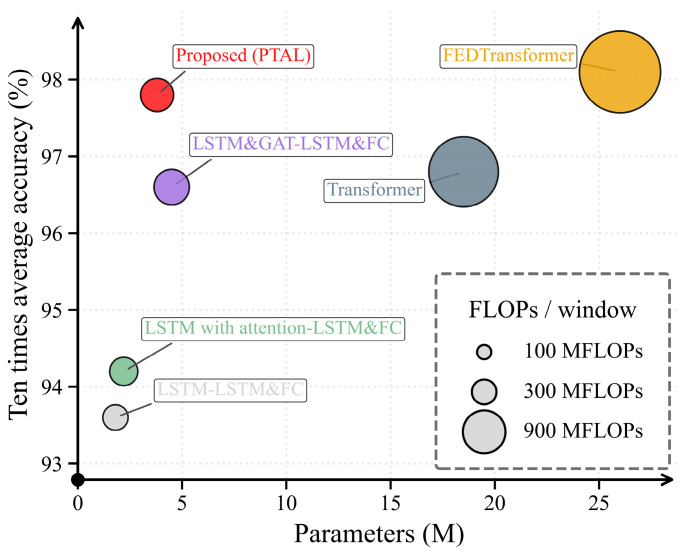
Accuracy–complexity trade-off (accuracy versus Params/FLOPs per window).

To validate the lightweight design under resource-constrained deployment requirements, we summarize the relationship between diagnostic performance and computational complexity using the accuracy–complexity trade-off plot in Figure 15, where complexity is characterized by model size and computation per input window. This visualization highlights the relative efficiency of PTAL compared with the baselines.

## 5. Conclusions

### 5.1. Summary and Key Insights

The proposed Physics-Topology-Anchored Learning (PTAL) framework demonstrates the critical value of physical inductive bias in complex systems such as RLREs. This study reveals the fundamental advantages of integrating deep learning with physical causal relationships:

(1) Physical Regularization. Physical inductive bias serves as a powerful regularizer that effectively constrains the model’s hypothesis space. Unlike purely data-driven architectures that require massive datasets to learn latent correlations, the “hard” structural anchoring in PTAL ensures high diagnostic stability even in data-scarce regimes.
(2) Compensatory Effect under Scarcity. The significant performance gap between PTAL (97.8%) and the base LSTM model (93.6%) under few-shot scenarios reveals that explicit physical topology compensates for the lack of fault samples by providing a deterministic roadmap for feature propagation.
(3) Efficiency Breakthrough. By introducing physical sparsity into the graph convolution logic, PTAL maintains high accuracy while significantly reducing computational overhead. Compared to large-scale Transformer-family models such as FEDTransformer (26.0 M parameters), PTAL achieves comparable performance with only 3.8 M parameters, proving its practical potential for on-board resource-constrained aerospace applications.

### 5.2. Limitations and Future Work

Despite its strong performance, the PTAL framework has certain limitations that define future research directions:

(1) Dependence on Prior Knowledge. The current framework relies on a predefined physical adjacency matrix, which assumes complete and accurate knowledge of system connectivity. In scenarios where physical models are incomplete or uncertain, the framework’s effectiveness may be affected.
(2) Adaptive Adjustment Mechanisms. Future research will explore “hybrid learned-physical graphs”. By introducing small, learnable residual branches (soft-anchoring) alongside the deterministic physical graph, the model could adaptively correct discrepancies between idealized physical models and actual physical processes.
(3) Scalability and Validation. Future work will also focus on extending PTAL to multi-engine systems and utilizing real-flight telemetry data to further enhance the framework’s robustness and reliability in extreme flight environments.

## Figures and Tables

**Table 1:** Prior relationships among parameters.

	Pc	Tc	fpbPc	lpftpPo	lpftpN	lpftpQ	hpftpPo	hpftpN	hpftpQ	ncP
Pc	0	1	0	1	0	1	0	1	1	1
Tc	1	0	0	0	1	0	0	0	0	1
fpbPc	0	0	0	0	0	0	1	1	0	0
lpftpPo	1	0	0	0	1	0	1	0	0	0
lpftpN	0	1	0	1	0	1	0	0	0	1
lpftpQ	1	0	0	0	1	0	0	0	0	0
hpftpPo	0	0	1	1	0	0	0	1	1	0
hpftpN	1	0	1	0	0	0	1	0	1	0
hpftpQ	1	0	0	0	0	0	1	1	0	0
ncP	1	1	0	0	1	0	0	0	0	0

**Table 2:** Computational complexity comparison of key components.

Model Component	Complexity	Rationale
Traditional GNN (dense learned graph)	$O(N^{2})$ (Dense Adj Matrix)	Learns many pairwise links.
PTAL (fixed physics graph)	O(E) (Sparse Physics Graph)	Aggregates only physical edges.
Self-attention (Transformer)	$O(L^{2})$	Quadratic in window length.
PTAL temporal module	O(L)	Linear recurrent update + lightweight weighting.

**Table 3:** The statistical results for the four models.

Models	LSTM-LSTM&FC	LSTM with Attention–LSTM&FC	LSTM&GAT-LSTM&FC	Transformer	FEDFormer	Proposed Model (PTAL)
Ten times average accuracy	93.61%	94.24%	96.67%	96.82%	98.14%	97.86%
Standard deviation	0.5536	0.5443	0.1740	0.1654	0.1297	0.1145
Precision	93.80%	94.50%	96.70%	97.10%	98.40%	98.10%
Recall	93.10%	93.80%	96.20	96.30%	97.60%	97.20%
F1-score	93.45%	94.15%	96.45	96.70%	98.00%	97.65%
*p*-value (vs. PTAL)	<0.001	<0.001	<0.001	<0.001	<0.001	-

Note: *p*-values are calculated via two-sample *t*-tests comparing each baseline to PTAL based on 10 independent runs.

**Table 4:** Learned correlations between sensor channels.

	Pc	Tc	fpbPc	lpftpPo	lpftpN	lpftpQ	hpftpPo	hpftpN	hpftpQ	ncP	F_Category
Pc	0.0000	0.2903	0.0000	0.2815	0.0000	0.2913	0.0000	0.3009	0.4398	0.2497	0.1213
Tc	0.3003	0.0000	0.0000	0.0000	0.2775	0.0000	0.0000	0.0000	0.0000	0.2209	0.1611
fpbPc	0.0000	0.0000	0.0000	0.0000	0.0000	0.0000	0.3077	0.3244	0.0000	0.0000	0.2432
lpftpPo	0.2148	0.0000	0.0000	0.0000	0.2096	0.0000	0.3160	0.0000	0.0000	0.0000	0.1932
lpftpN	0.0000	0.3231	0.0000	0.3492	0.0000	0.3708	0.0000	0.0000	0.0000	0.2686	0.1289
lpftpQ	0.2223	0.0000	0.0000	0.0000	0.2204	0.0000	0.0000	0.0000	0.0000	0.0000	0.1898
hpftpPo	0.0000	0.0000	0.3347	0.1864	0.0000	0.0000	0.0000	0.1234	0.1871	0.0000	0.0879
hpftpN	0.0648	0.0000	0.3328	0.0000	0.0000	0.0000	0.1251	0.0000	0.1878	0.0000	0.0876
hpftpQ	0.0648	0.0000	0.0000	0.0000	0.0000	0.0000	0.1262	0.1260	0.0000	0.0000	0.1157
ncP	0.0659	0.1956	0.0000	0.0000	0.1464	0.0000	0.0000	0.0000	0.0000	0.0000	0.1529
F_Category	0.1213	0.1611	0.2432	0.1932	0.1289	0.1898	0.0879	0.0876	0.1157	0.1529	0.0000

## Data Availability

Data will be made available on request.
